# Carbimazole-Induced Agranulocytosis in a Previously Stable Patient: A Case Report and Literature Review

**DOI:** 10.7759/cureus.24115

**Published:** 2022-04-13

**Authors:** Zahid Khan, Waleed Afifi, Syed Aun Muhammad, Vinod Warrier

**Affiliations:** 1 Cardiology and General Medicine, Barking, Havering and Redbridge University Hospitals NHS Trust, London, GBR; 2 Cardiology, Royal Free Hospital, London, GBR; 3 Internal Medicine, Mid and South Essex NHS Foundation Trust, Southend-on-Sea, GBR; 4 Cardiology, Mid and South Essex NHS Foundation Trust, Southend-on-Sea, GBR

**Keywords:** thyroid stimulating hormone tsh, atypical chest pain, ent procedures, heart palpitations, antithyroid antibodies, antithyroid medications, propranolol, hyperthyroidism, drug induced agranulocytosis

## Abstract

Carbimazole-induced agranulocytosis is a rare condition. A 48-year-old female patient with a history of hyperthyroidism for several years presented with generalized fatigue and chest pain associated with palpitations; the patient was stable on carbimazole therapy. She was previously on carbimazole 15 mg once daily (OD) and, three months later, was reduced to 10 mg OD as symptoms were controlled. She was taking propranolol, and it was stopped as she was not having any palpitations. This time, however, she presented mainly with palpitations at night associated with chest pain and generalized fatigue ongoing for the last one week. Her laboratory results showed agranulocytosis with reduced white cell count and neutrophil count in the absence of any evidence of infection. Her carbimazole was stopped, and the patient was referred to ear, nose, and throat surgeons for consideration of thyroidectomy. The patient underwent subtotal thyroidectomy, and her symptoms resolved following surgery.

## Introduction

Carbimazole is a prodrug used to treat patients with hyperthyroidism through conversion to its active metabolite form (methimazole) [1]. It may take four to eight weeks for symptoms to completely resolve, and typical maintenance dose ranges from 5 to 30 mg/day [1]. Patients on higher doses of carbimazole (> 30 mg/day) for more than two months and aged > 40 years are at risk of developing agranulocytosis [1,2]. The incidence of this carbimazole-induced agranulocytosis has been reported to range from 0.3% to 0.6% and is associated with a very high mortality rate of up to 21.5% [3,4]. Previous studies show that agranulocytosis from carbimazole occurs in patients usually in the first two to three months, and it usually happens in patients on higher doses [5]. Another study has reported the incidence of agranulocytosis from carbimazole up to a year after the initiation of the therapy, and it has reported a slightly lower incidence of the condition ranging from 0.17% to 0.36% [6]. The incidence of agranulocytosis was 6.4 times higher in patients aged > 40, although few cases have also been reported in younger patients [6].

## Case presentation

**Figure 1:**
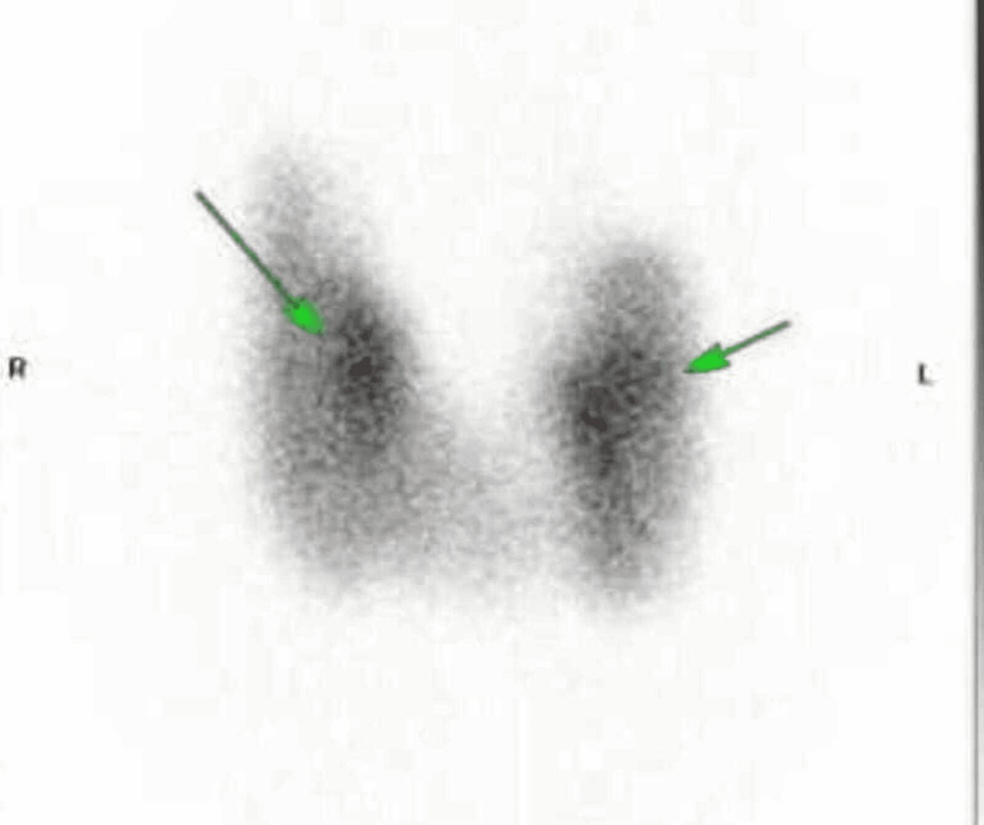
Radioactive iodine uptake (RAIU) test shows large right and left thyroid lobes (arrows) Right (R) thyroid lobe is larger than left (L).

A 48-year-old patient presented with palpitations associated with chest pain at nighttime that was waking her up for the last one week. She had a past medical history (PMH) of hyperthyroidism diagnosed in 2015 which resolved without treatment. She was commenced on carbimazole 15 mg OD and propranolol 10 mg BD initially in late 2020. Her carbimazole was reduced to 10 mg OD in early 2021 as her thyroid functions tests (TFTs) improved, and she remained symptom free. Her thyroid-stimulating hormone (TSH) was <0.01 mU/L and free T4 was 50.1 pmol/L in late 2020 when she was started on treatment, and TFTs improved after two months with TSH level remaining <0.01, free T4 14.4 pmol/L, and free T3 7.6 pmol/L. Radioactive iodine uptake (RAIU) test also known as thyroid uptake scan showed large right and left thyroid lobes, where the right thyroid lobe is larger than the left consistent with mild thyrotoxicosis as shown in Figure 1.

She continued with carbimazole 10 mg OD; however, propranolol was stopped in early 2022 as she did not have any palpitations or weight loss. Her TFTs showed further improvement with TSH <0.01 mU/L, free T4 13.4 pmol/L, and free T3 6.7 pmol/ as shown in Table 1.

**Table 1:** Laboratory results for a patient showing thyroid hormone and antibodies trend TSH: thyroid-stimulating hormone.

Investigation/Test	Range	2020	2021	2022
White cell count	4.5-11.0 × 10^9^/L	7.5	3.7	2.8
Neutrophil	1.8-7.5 × 10^9^/L	6.5	6.8	1.2
Mean corpuscular volume	80-100 fL	78	76	74
Platelet count	150-400 × 10^9^/L	203	198	196
C-reactive protein	<5 mg/L	<5	7	<5
Urea	2.5-7.8 mmol/L	5.4	5.8	3.9
Creatinine	45-89 µmol/L	67	65	63
Potassium	3.5-5.3 mmol/L	4.3	4.1	4.2
Sodium	133-146 mmol/L	135	138	137
TSH receptor antibody	0-0.9 IU/L	3.1	2.8	2.3
Antithyroid peroxidase	<9 IU/mL	962	858	835
Thyroid-stimulating hormone (TSH)	0.3-5.0 mU/L	<0.01	<0.01	<0.01
Free T4	7.9-16.0 pmol/L	15.6	14.4	13.6
Free T3	3.8-6.0 pmol/L	8.9	7.6	6.7

She had all three COVID-19 vaccines, and her last vaccine was more than four months ago. She denied any sore throat, fever, and shortness of breath. She was having irregular palpitations at night for the past one week. She was also having generalized fatigue and mild upper back pain. The patient’s clinical examination was unremarkable, chest was clear, and heart sounds were normal. Her laboratory results showed a white cell count (WCC) of 2.8 × 10^9^/L and neutrophil count of 1.2 × 10^9^/L, and other blood tests were normal. Her previous white cell count was 3.7 × 10^9^/L eight months back, and neutrophil count was normal. Her chest radiograph was normal, and urine dip was negative for nitrites.

The patient was diagnosed with carbimazole-induced agranulocytosis, and it was stopped. The patient was reviewed by an endocrine specialist and was referred to ear, nose, and throat (ENT) surgeons for consideration of thyroidectomy. The patient underwent subtotal thyroidectomy, and her thyroid function tests returned to normal a month after the procedure. She will be followed up for a longer time period in the outpatient clinic due to the risk of developing hypothyroidism.

## Discussion

Carbimazole is a prodrug that gets converted to its active metabolite (methimazole) used for the treatment of hyperthyroidism alone or in combination with other medications. The common clinical features of carbimazole induced agranulocytosis include fever (92%), sore throat (85%), painful mouth ulcer (15%), ulcer (8%), and reduced immune response making individuals prone to infections [3]. Agranulocytosis and hepatotoxicity are two very rare but life-threatening side effects of antithyroid therapy (ATT) [3]. Signs may vary, and the most common signs in patients with agranulocytosis include ulcerative changes in the upper respiratory tract mucous membranes, ulcerative-necrotic tonsillitis, gastrointestinal side effects, fever, regional lymphadenopathy, and fungal infections [3]. Patients with agranulocytosis mostly present with fever and sore throat, and a study reported that even close monitoring of the blood count may not prevent its occurrence [7,8].

Antithyroid drugs (ATDs), particularly propylthiouracil and carbimazole, can cause hematological effects such as mild leukopenia, agranulocytosis, and aplastic anemia, although the reported incidence is rare [6]. These effects can result from direct toxic effects of the medications and immunological reactions, and ATDs affect oxygen and glucose utilization in cells through oxidized metabolites after penetrating the bone marrow. It may take two to eight weeks for the agranulocytosis to appear in patients after being started on ATD [6,9]. The WCC can return to normal in patients over one to two weeks’ time period after discontinuation of the offending medication, but it can take up to one to eight weeks sometimes [9]. Recovery is usually quick if there are enough myeloid precursors cells present in the bone marrow; however, it may be prolonged in the presence of granulocyte precursor aplasia [9].

A study from 1983 based on patients with hyperthyroidism reported that patients aged > 40 years and on higher doses of methimazole (> 30 mg daily) were associated with an increased risk of agranulocytosis [10]. This diagnosis is however not applicable to patients on chemotherapy and/or patients with chronic autoimmune neutropenia. Prognosis depends on age, comorbidity, duration of agranulocytosis, and its severity and can lead to adverse outcome in patients with severe agranulocytosis if timely intervention is not made including stopping the offending drug and commencing intravenous antibiotics [4,10]. ATDs are increasingly used in patients with hyperthyroidism which is a common endocrine disorder affecting women more than men with a reported prevalence of 1%-2% and 0.1%-0.2% in women and men, respectively [8].

In a retrospective study, 18 Grave's disease patients with ATD-induced agranulocytosis were > 20 years of age, and no correlation was found between age and the development of agranulocytosis. Ninety-five percent of patients developed ATD-induced agranulocytosis within two to 12 weeks of starting ATD treatment, and the condition was found to be dose dependent; it developed abruptly in patients, and weekly blood tests failed to predict the occurrence of the disease. The study reported that granulocyte-macrophage colony-stimulating factor (GM-CSF) was associated with an increase in granulocyte count; however, glucocorticoid treatment was found to be ineffective [11]. Similar findings have been reported in other studies, and GM-CSF was found to enhance the recovery of the peripheral blood granulocyte lineage, resulting in faster normalization of peripheral granulocyte count which in turn reduces the risk of fatal bacterial complications [12].

Patients undergoing subtotal thyroidectomy are at increased risk of developing hypothyroidism and should have regular follow-ups. Patients who underwent surgical thyroidectomy were followed up for 54.6 months on average, and 83.9% of patients developed hypothyroidism 1.3 months following surgery; 10% of patients had persistent hyperthyroidism, and euthyroid status was established in only 6.5% of patients. The median time for 10% of patients with persistent hyperthyroidism was 9.7 months (range: 1.1-86.3 months) to achieve euthyroid status [13].

## Conclusions

In conclusion, hyperthyroidism is a common condition treated mostly with antithyroid medications. This patient has been stable on treatment for over a year and did not develop typical symptoms of agranulocytosis such as sore throat, fever, or lymphadenopathy. It is important to routinely monitor blood count in patients on antithyroid medications, particularly those who are aged > 40 years and on higher doses (> 30 mg/day) for a longer duration. Antithyroid medications should immediately be stopped in these patients, and infections should be ruled out. These patients need long-term follow-up after surgery, and most patients will require thyroid hormone replacement.
