# Correction: Carbimazole-Induced Agranulocytosis in a Previously Stable Patient: A Case Report and Literature Review

**DOI:** 10.7759/cureus.c96

**Published:** 2023-01-17

**Authors:** Zahid Khan, Waleed Afifi, Syed Aun Muhammad, Vinod Warrier

**Affiliations:** 1 Cardiology and General Medicine, Barking, Havering and Redbridge University Hospitals NHS Trust, London, GBR; 2 Cardiology, Royal Free Hospital, London, GBR; 3 Internal Medicine, Mid and South Essex NHS Foundation Trust, Southend-on-Sea, GBR; 4 Cardiology, Mid and South Essex NHS Foundation Trust, Southend-on-Sea, GBR

This article has been corrected at the request of the author due to presence of an error in the Discussion section which was noticed by a reader. The second sentence of the first paragraph in the Discussion section has been corrected as the original version incorrectly referred to "side effects", when in reality they are "clinical features".

Original (incorrect): The common side effects of carbimazole include fever (92%), sore throat (85%), painful mouth ulcer (15%), ulcer (8%), and reduced immune response making individuals prone to infections.

Corrected: The common clinical features of carbimazole induced agranulocytosis include fever (92%), sore throat (85%), painful mouth ulcer (15%), ulcer (8%), and reduced immune response making individuals prone to infections.

 

